# Supplementary material for: Half-Wave Potentials and In Vitro Cytotoxic Evaluation of 3-Acylated 2,5-Bis(phenylamino)-1,4-benzoquinones on Cancer Cells
Source: Molecules. 2019 May 8;24(9):1780. doi: 10.3390/molecules24091780 (PMC6539005; doi:10.3390/molecules24091780)
Supplement: Supplementary file 1 [file molecules-24-01780-s001.pdf]

Supplementary Materials

3-Butiryl-2,5-bis(phenylamino)-1,4-benzoquinone **2b** ^{1}**H NMR,** ^{13}**C NMR and HMBC**

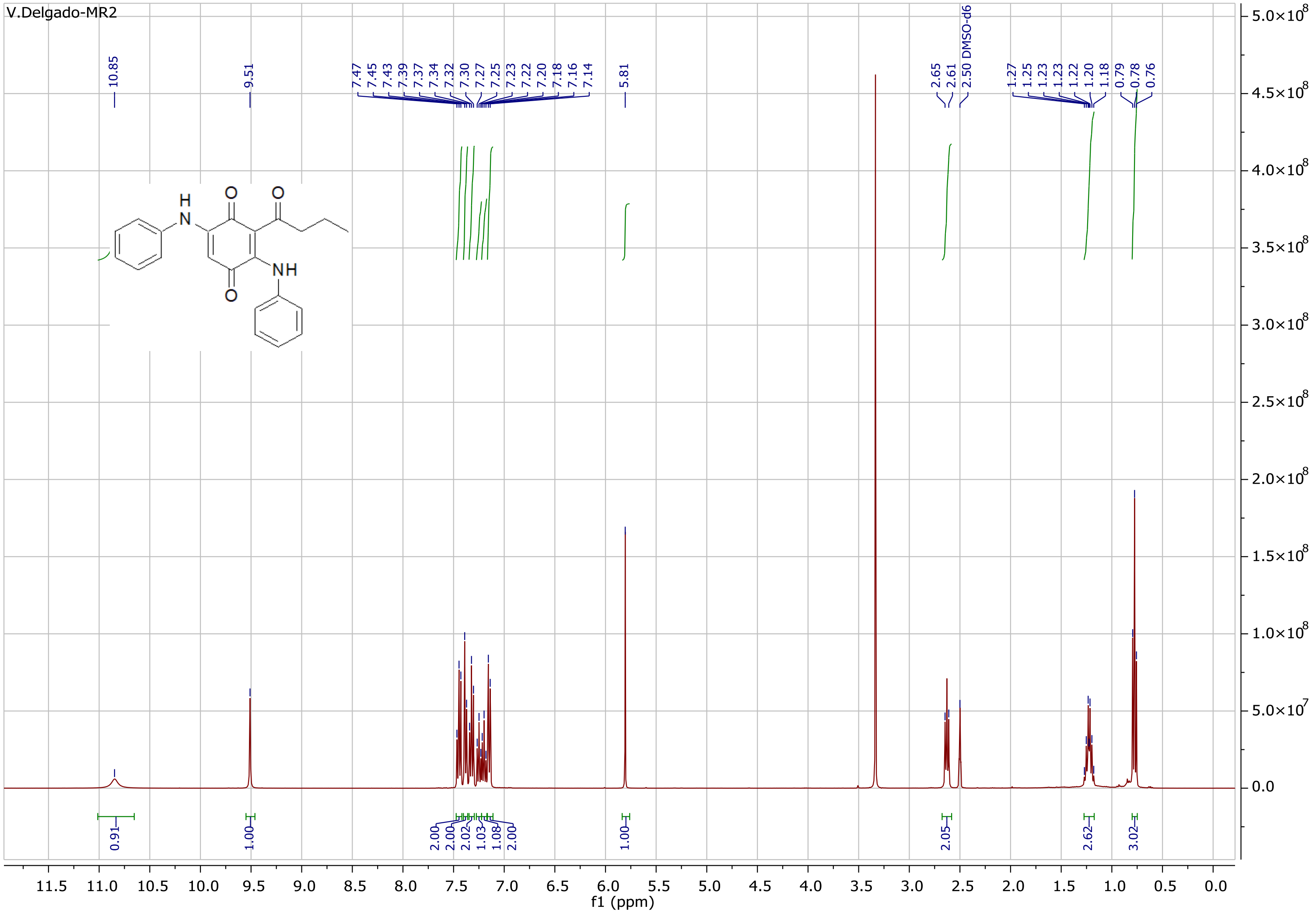

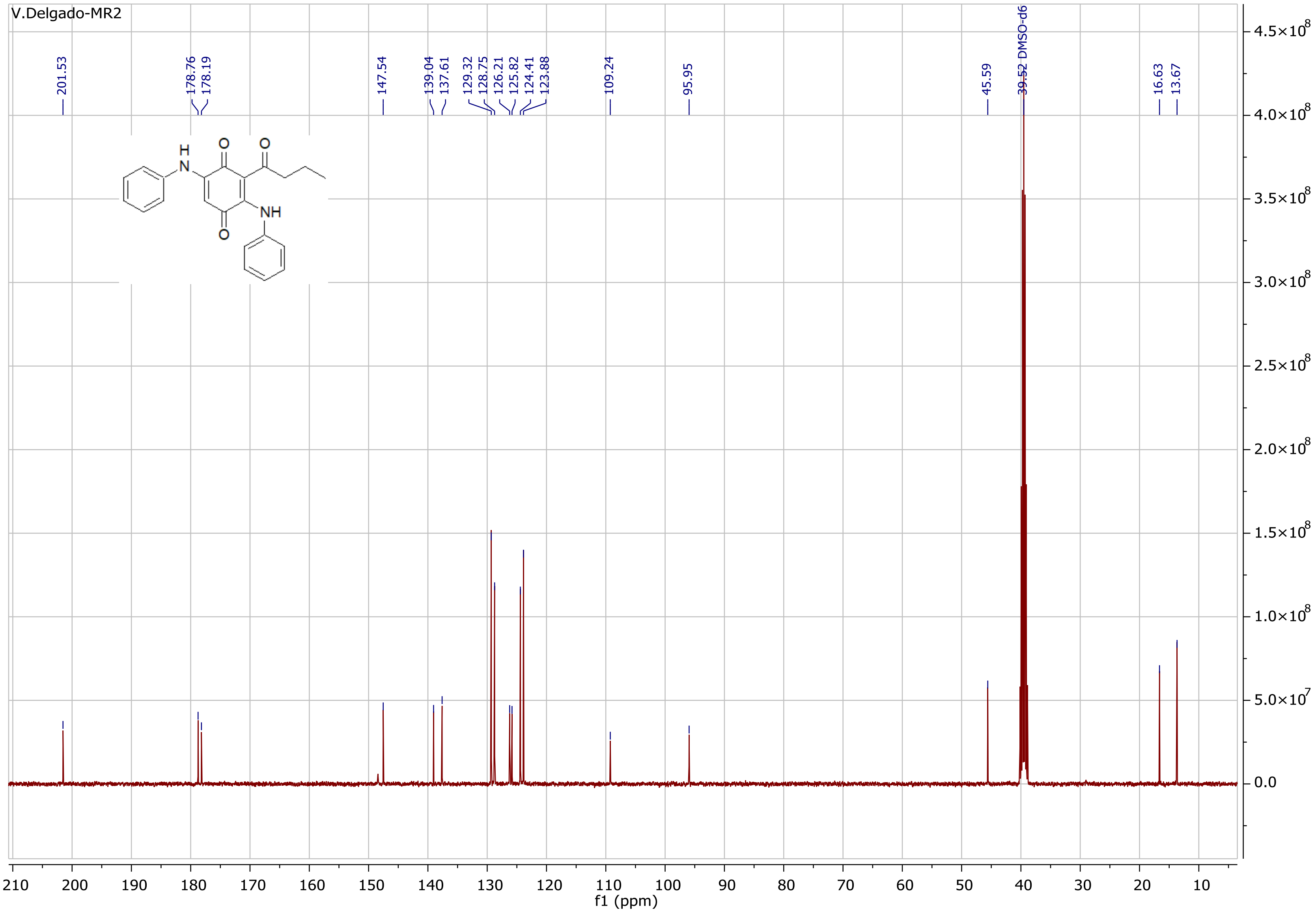

V.Delgado-MR2
201.53
178.76
178.19
147.54
139.04
137.61
129.32
128.75
126.21
125.82
124.41
123.88
109.24
95.95
45.59
39.52 DMSO-d6
16.63
13.67
H
N
O
O
NH
O
4.5×10^8
4.0×10^8
3.5×10^8
3.0×10^8
2.5×10^8
2.0×10^8
1.5×10^8
1.0×10^8
5.0×10^7
0.0
210
200
190
180
170
160
150
140
130
120
110
100
90
80
70
60
50
40
30
20
10
f1 (ppm)

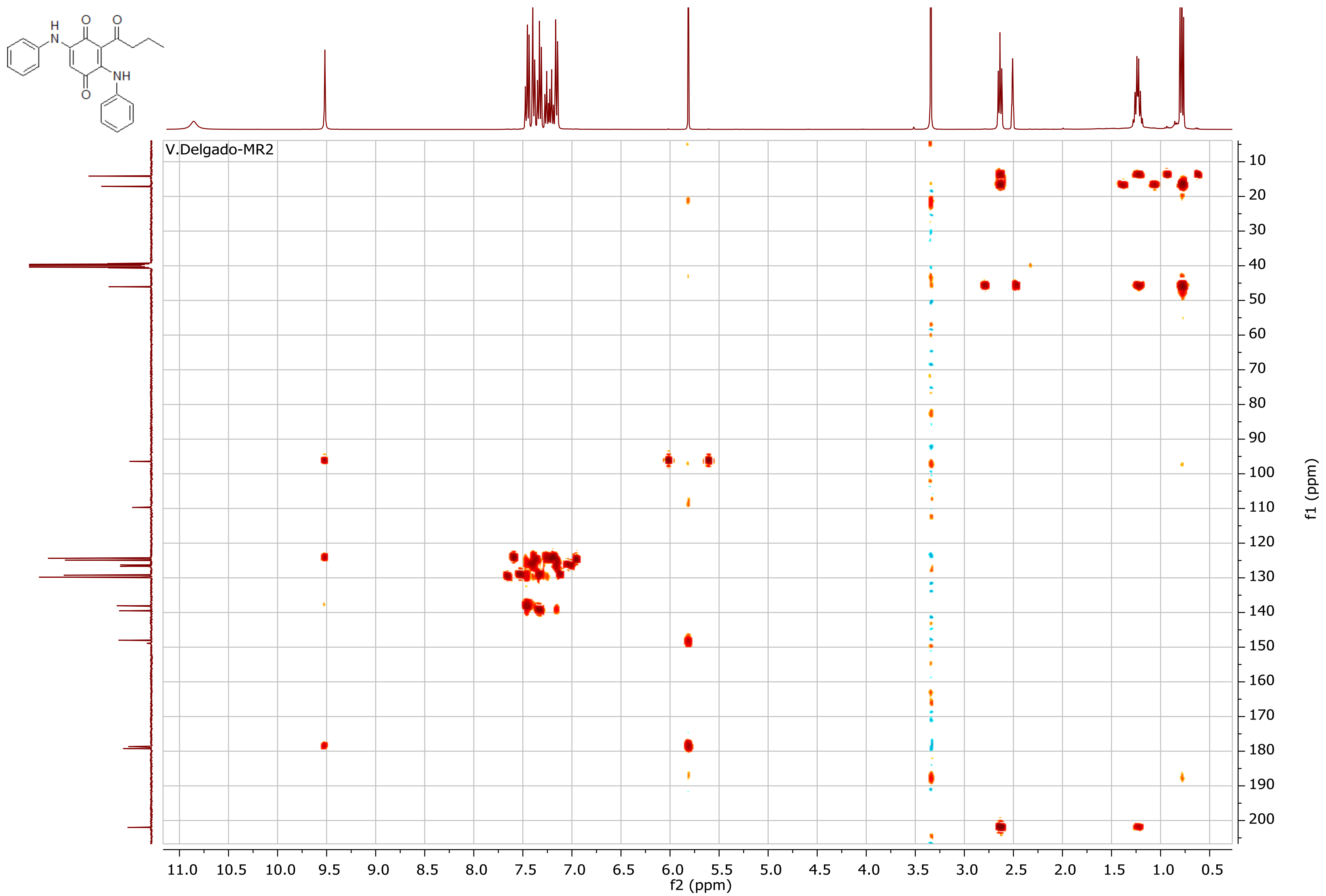

V.Delgado-MR2
f2 (ppm)
f1 (ppm)

3-(Furan-2-carbonyl)-2,5-bis(phenylamino)-1,4-benzoquinone **2g** **^{1}H NMR, ^{13}C NMR and HMBC**

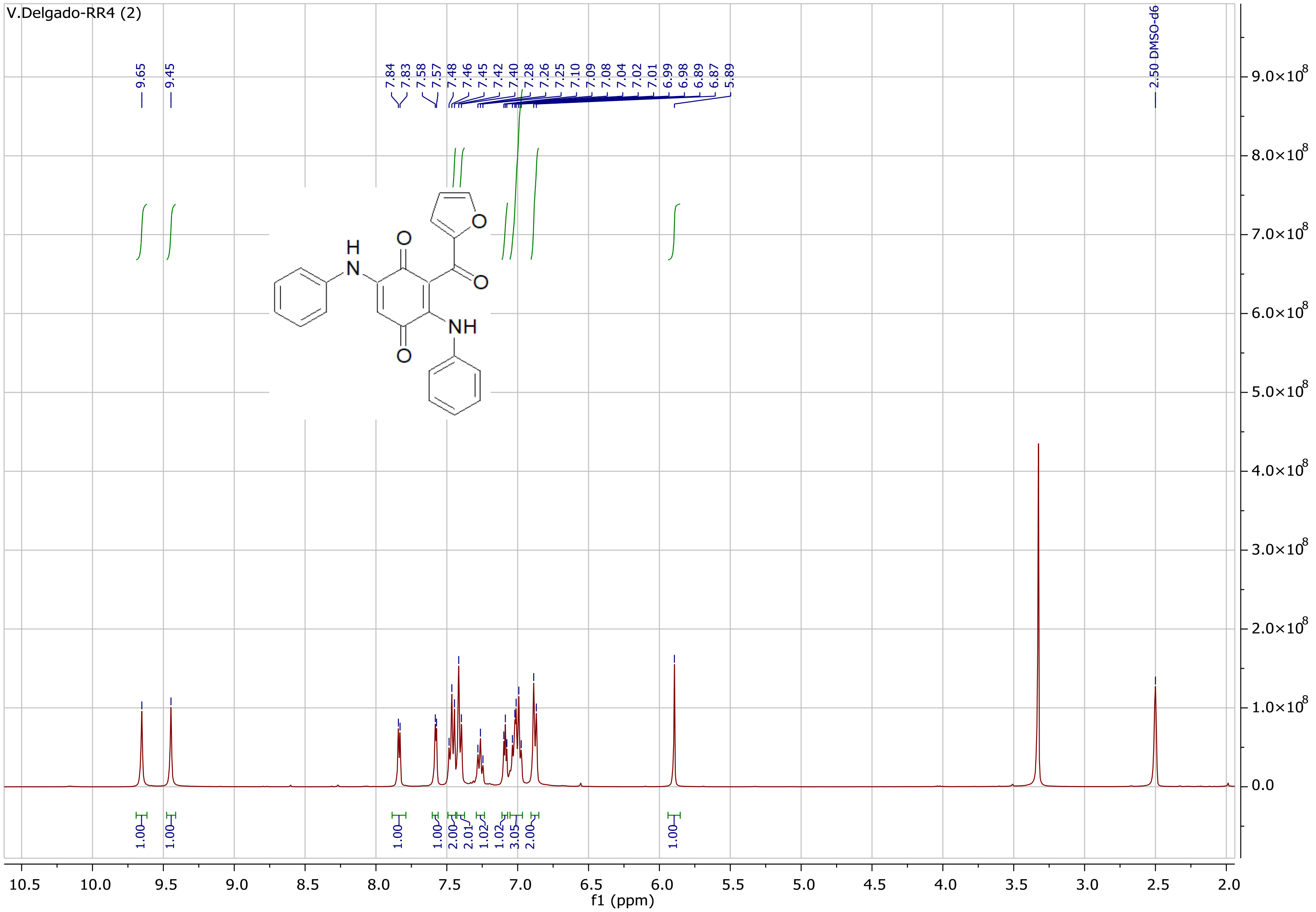

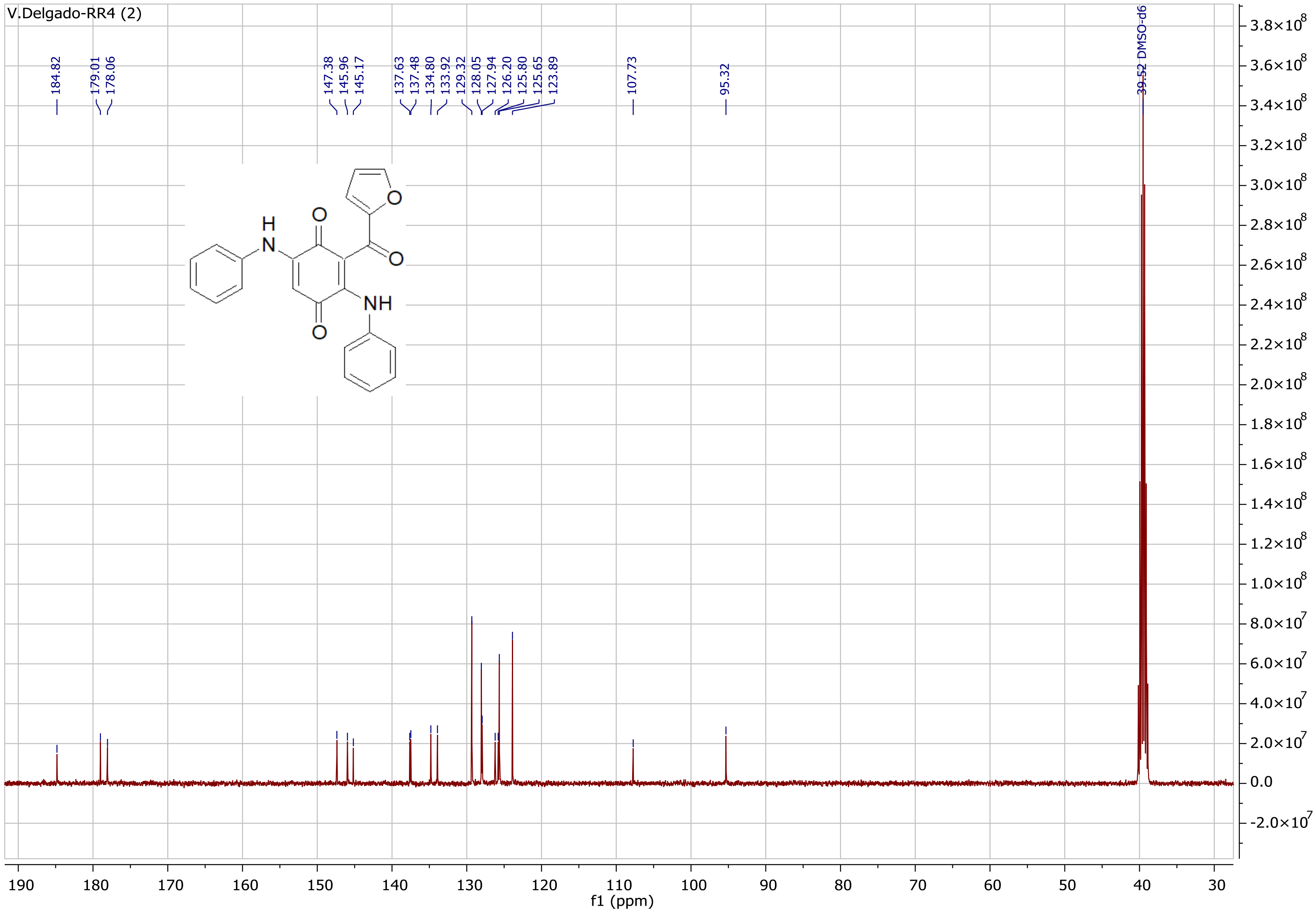

V.Delgado-RR4 (2)
184.82
179.01
178.06
147.38
145.96
145.17
137.63
137.48
134.80
133.92
129.32
128.05
127.94
126.20
125.80
125.65
123.89
107.73
95.32
39.52 DMSO-d6
f1 (ppm)

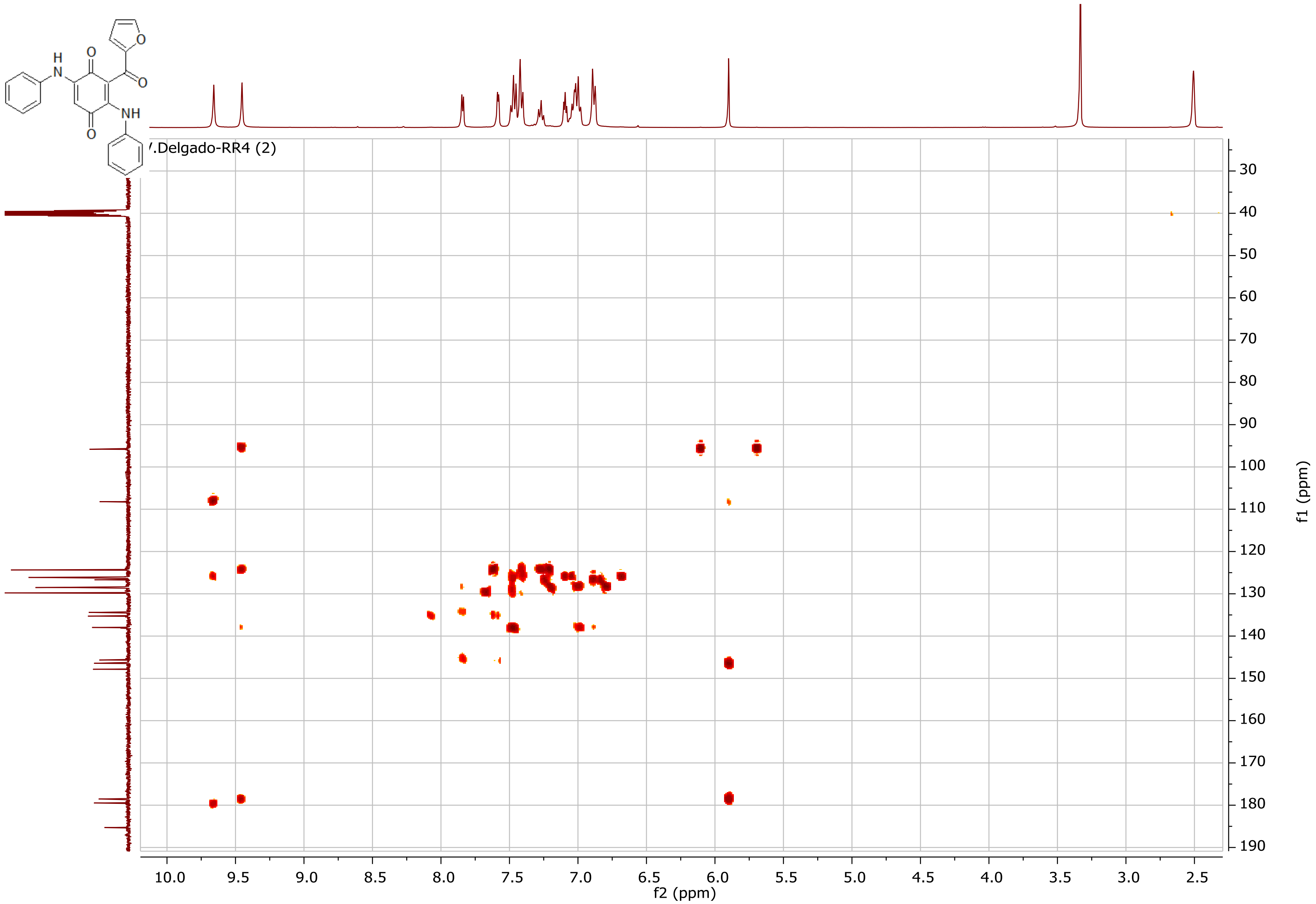

H
N
O
O
O
O
NH
/.Delgado-RR4 (2)
10.0
9.5
9.0
8.5
8.0
7.5
7.0
6.5
6.0
5.5
5.0
4.5
4.0
3.5
3.0
2.5
f2 (ppm)
30
40
50
60
70
80
90
100
110
120
130
140
150
160
170
180
190
f1 (ppm)

3-(Thiophen-2-carbonyl)-2,5-bis(phenylamino)-1,4-benzoquinone **2h** **^{1}H NMR, ^{13}C NMR and HMBC**

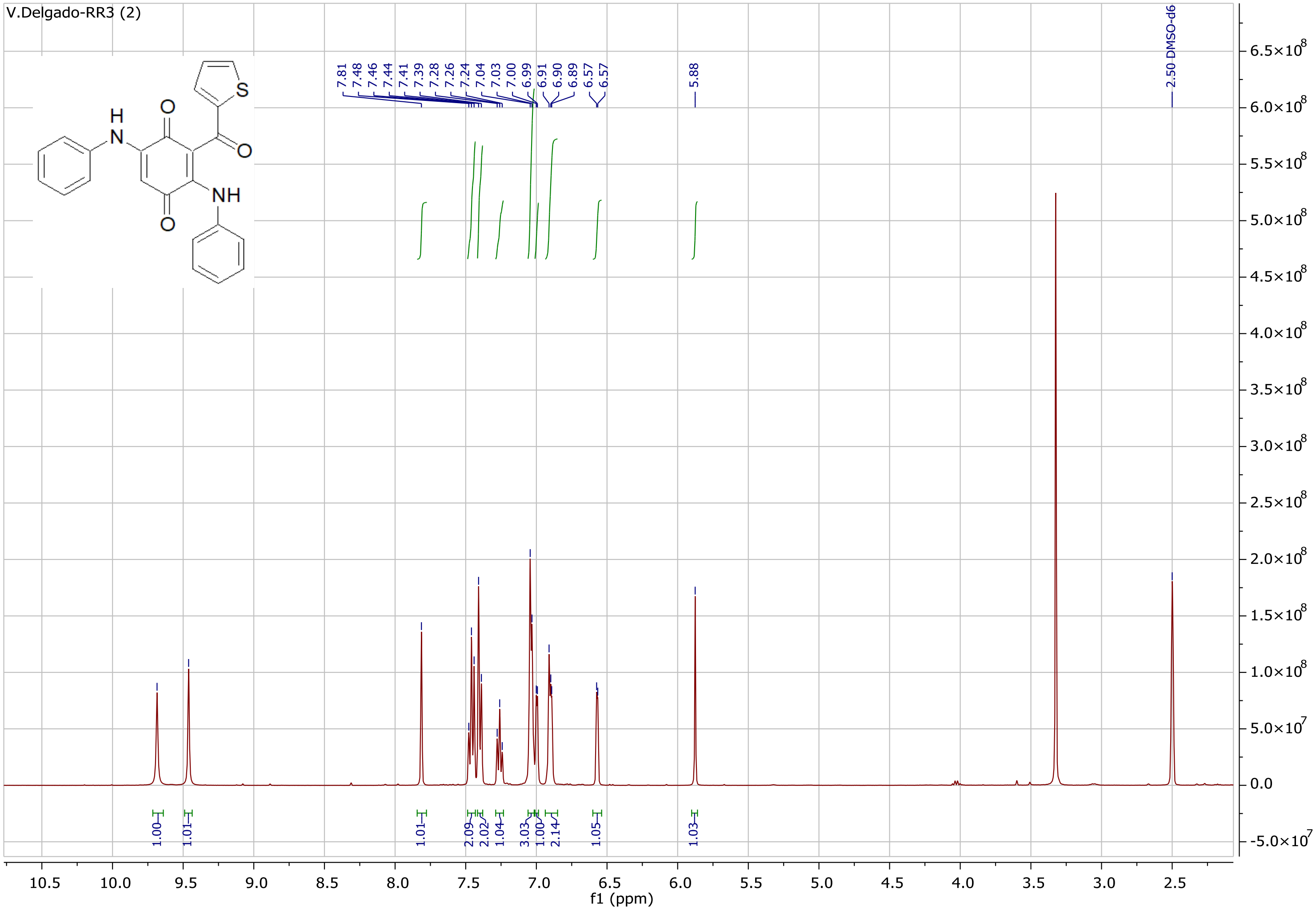

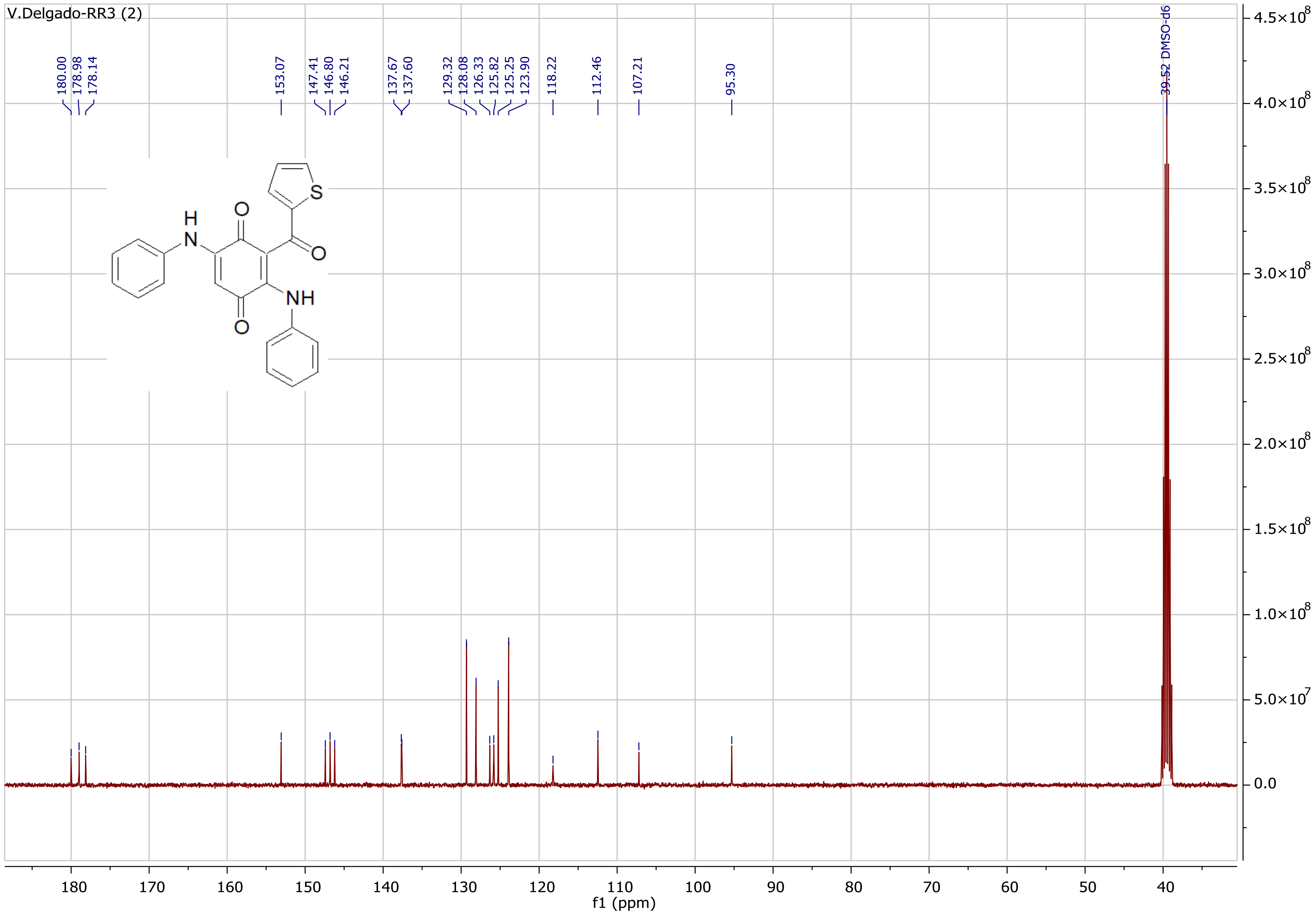
V.Delgado-RR3 (2)
180.00
178.98
178.14
153.07
147.41
146.80
146.21
137.67
137.60
129.32
128.08
126.33
125.82
125.25
123.90
118.22
112.46
107.21
95.30
39.52 DMSO-d6
H
N
O
S
O
NH
O
4.5×10^8
4.0×10^8
3.5×10^8
3.0×10^8
2.5×10^8
2.0×10^8
1.5×10^8
1.0×10^8
5.0×10^7
0.0
180
170
160
150
140
130
120
110
100
90
80
70
60
50
40
f1 (ppm)

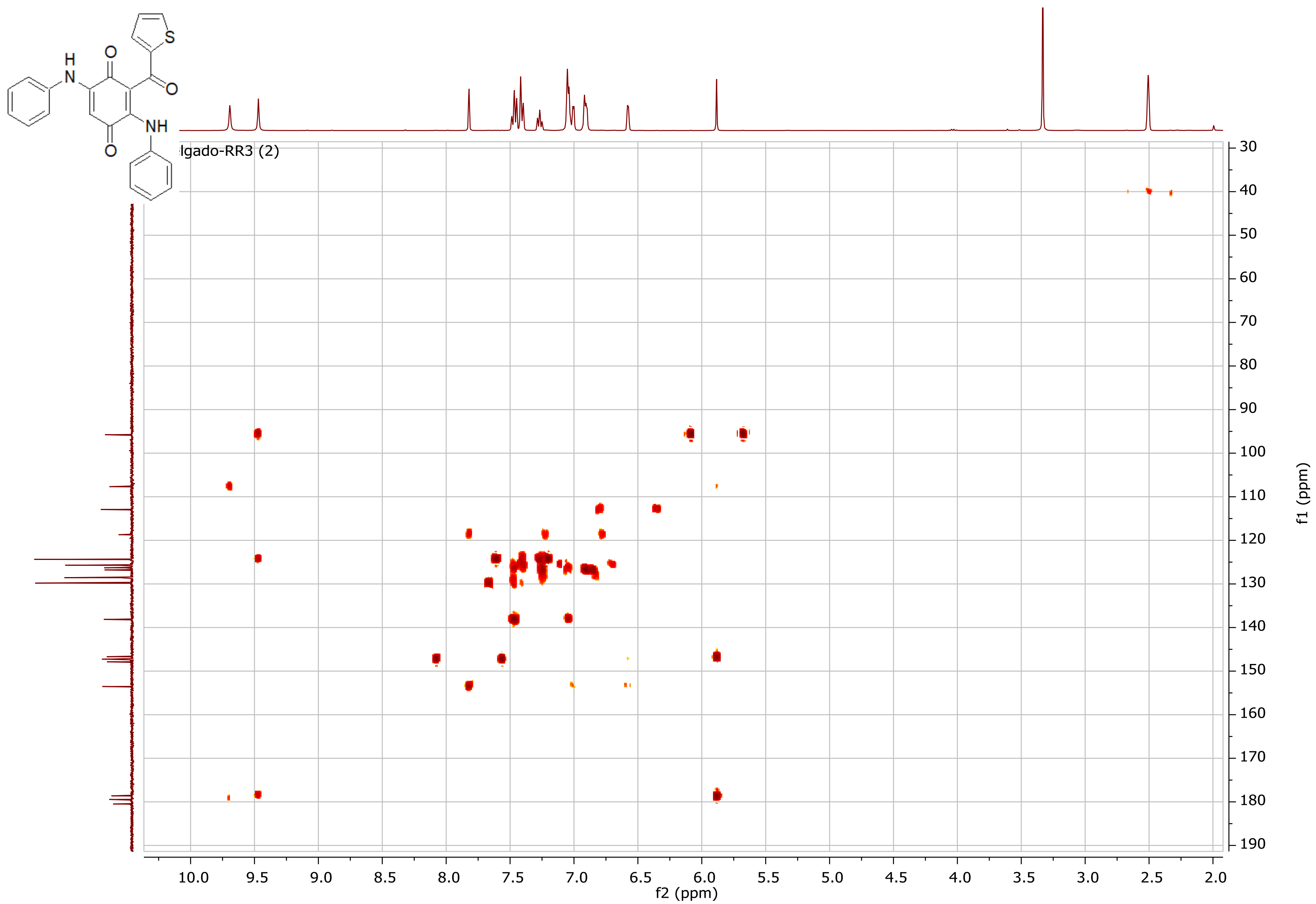

lgado-RR3 (2)
f2 (ppm)
f1 (ppm)

3-(4-Methoxybenzoyl)-2,5-bis-[(3,4,5-trimethoxyphenyl)amino]-1,4-benzoquinone **2j** **^{1}H NMR and ^{13}C NMR**

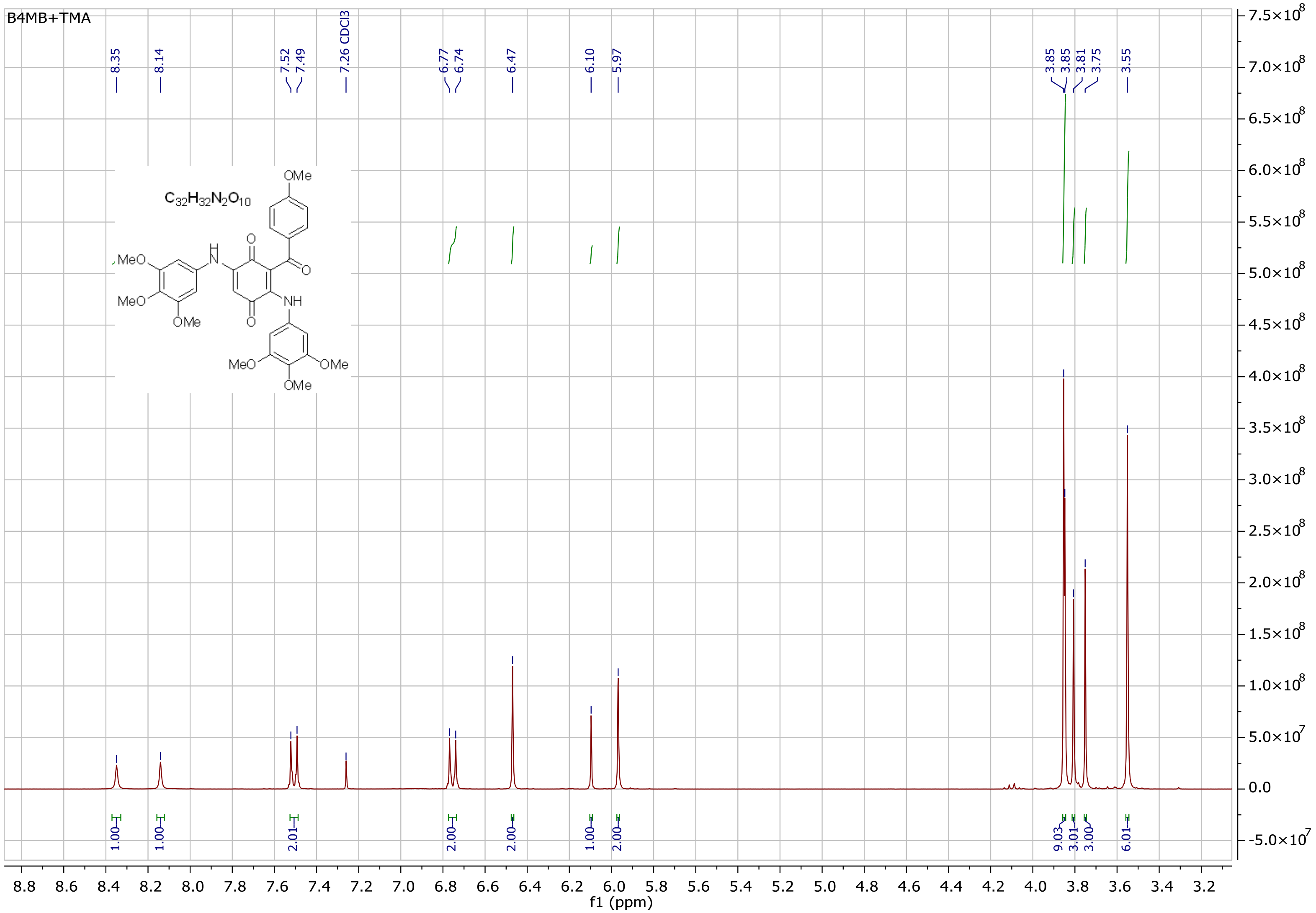

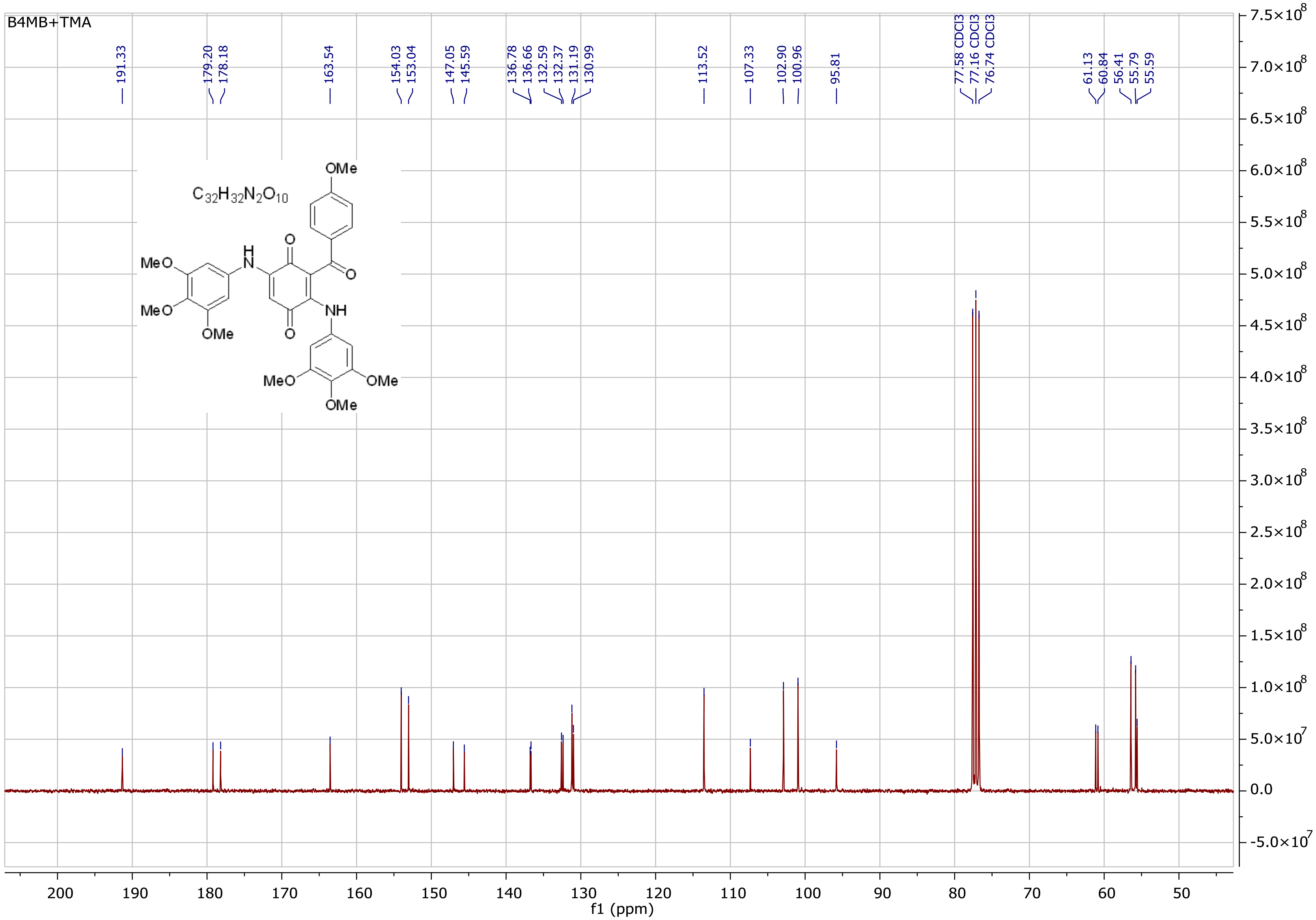

B4MB+TMA
191.33
179.20
178.18
163.54
154.03
153.04
147.05
145.59
136.78
136.66
132.59
132.37
131.19
130.99
113.52
107.33
102.90
100.96
95.81
77.58 CDCl3
77.16 CDCl3
76.74 CDCl3
61.13
60.84
56.41
55.79
55.59
$C_{32}H_{32}N_2O_{10}$
OMe
MeO
MeO
OMe
H
N
O
O
NH
O
MeO
OMe
OMe
f1 (ppm)

3-(Thiophen-2-carbonyl)-2,5-bis-[(3,4,5-trimethoxyphenyl)amino]-1,4-benzoquinone **2m** **^{1}H NMR, ^{13}C NMR and HMBC**

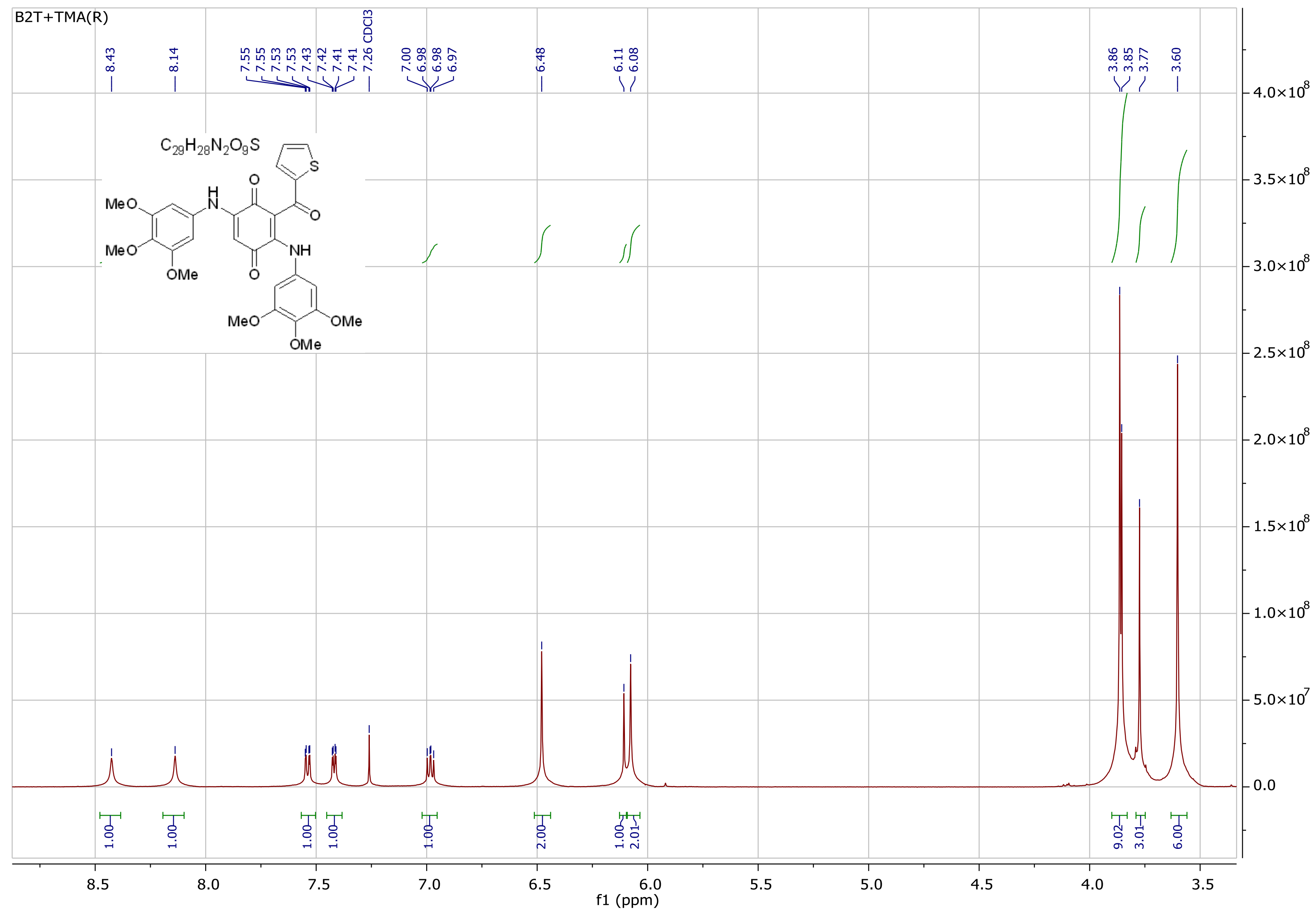

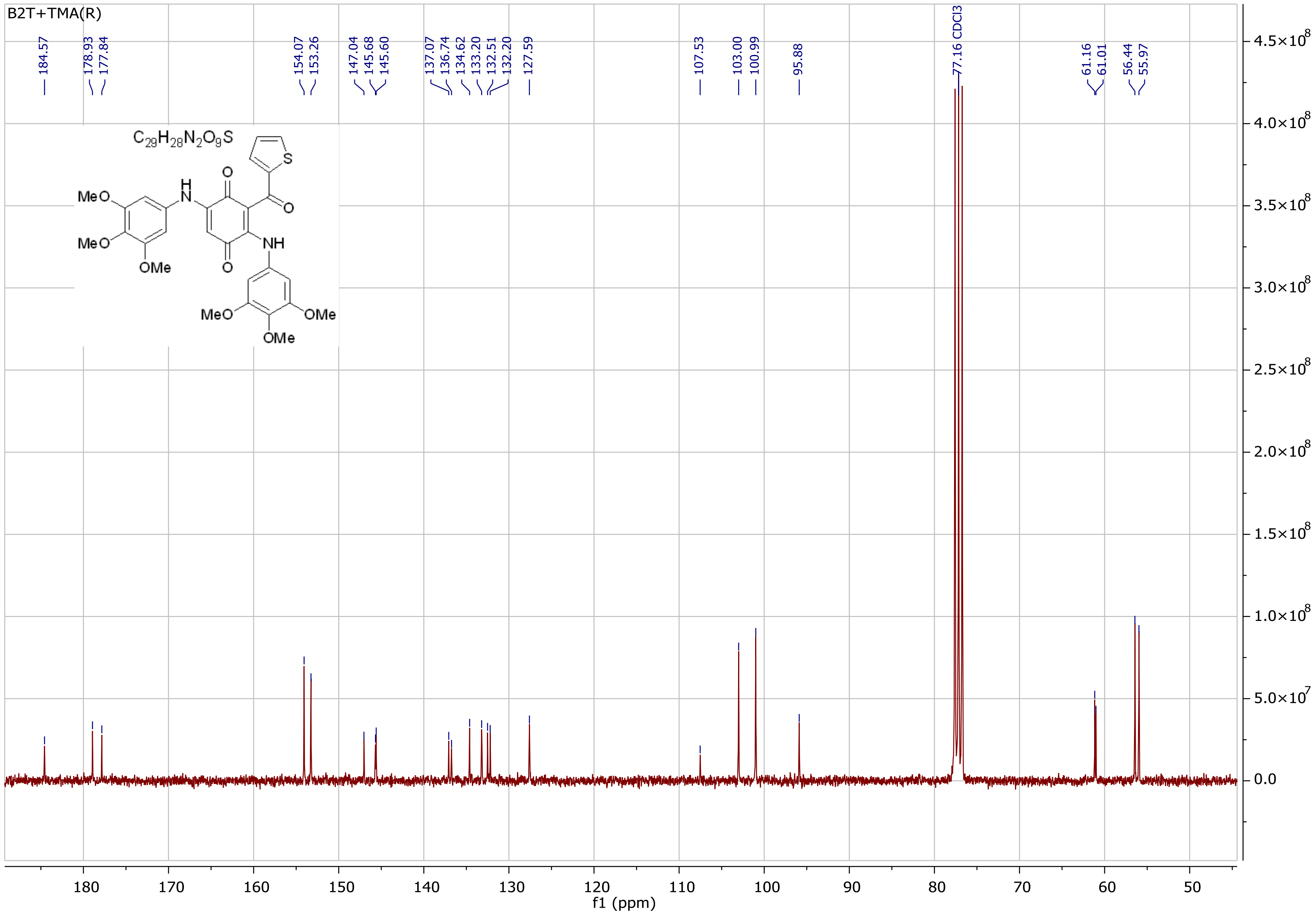

B2T+TMA(R)
184.57
178.93
177.84
154.07
153.26
147.04
145.68
145.60
137.07
136.74
134.62
133.20
132.51
132.20
127.59
107.53
103.00
100.99
95.88
77.16 CDCl3
61.16
61.01
56.44
55.97
C29H28N2O9S
MeO
MeO
OMe
H
N
O
O
S
O
NH
MeO
OMe
OMe
f1 (ppm)

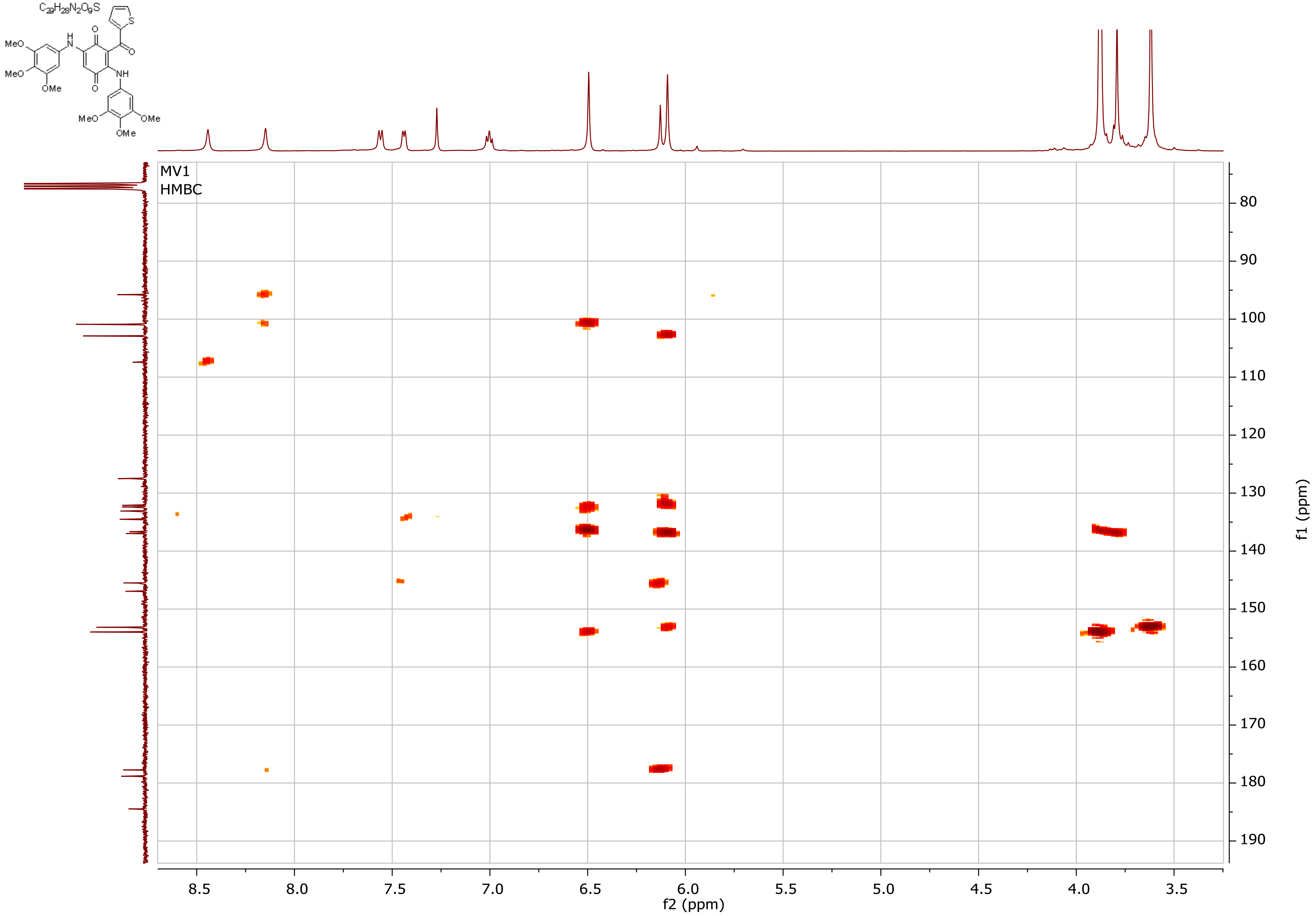

C29H28N2O9S
MeO
MeO
OMe
MeO
OMe
OMe
NH
MV1
HMBC
f2 (ppm)
f1 (ppm)

3-(4-Methoxybenzoyl)-2,5-bis-[(4-fluorophenyl)amino]-1,4-benzoquinone **2ñ** **^{1}H NMR and ^{13}C NMR**

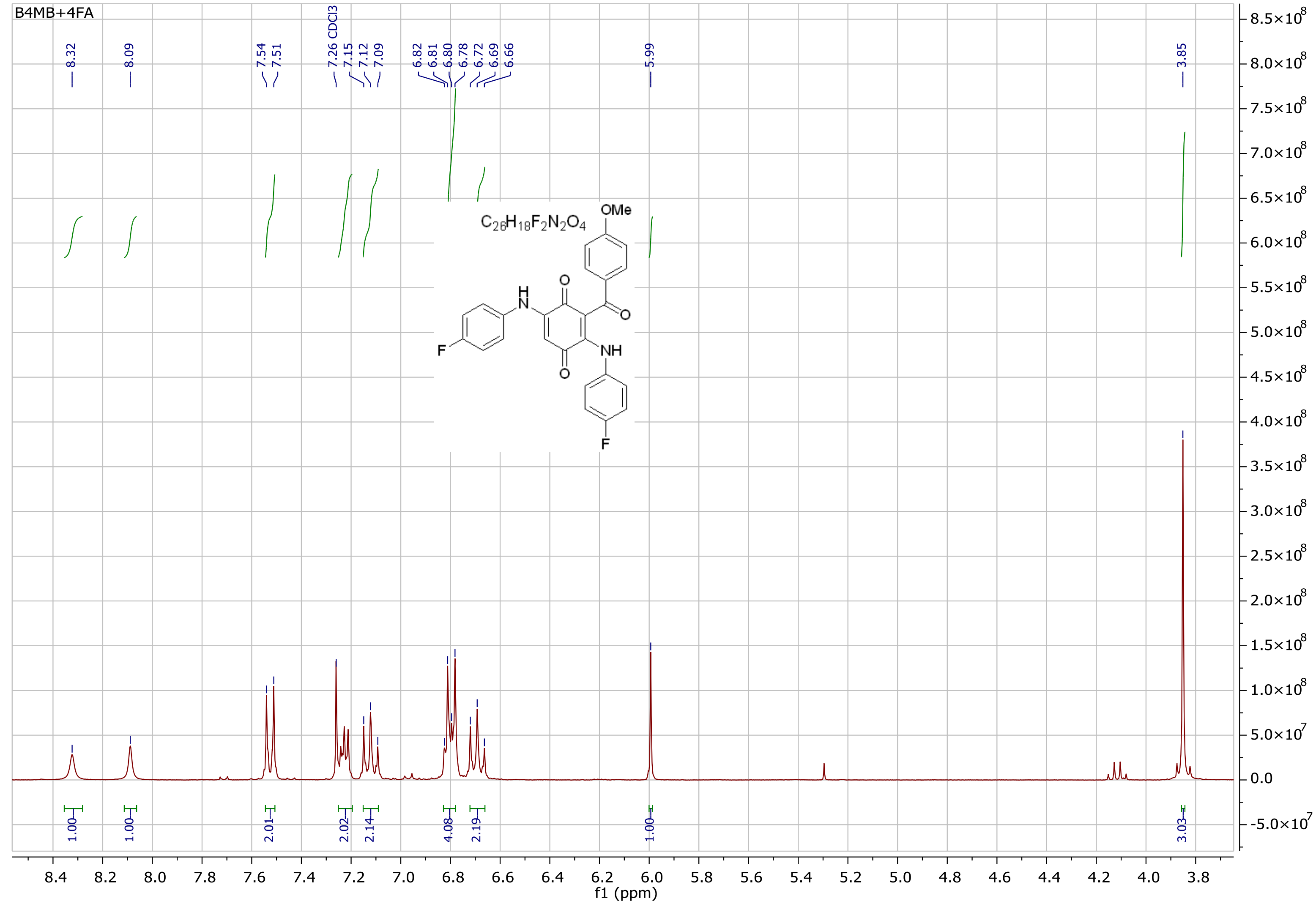

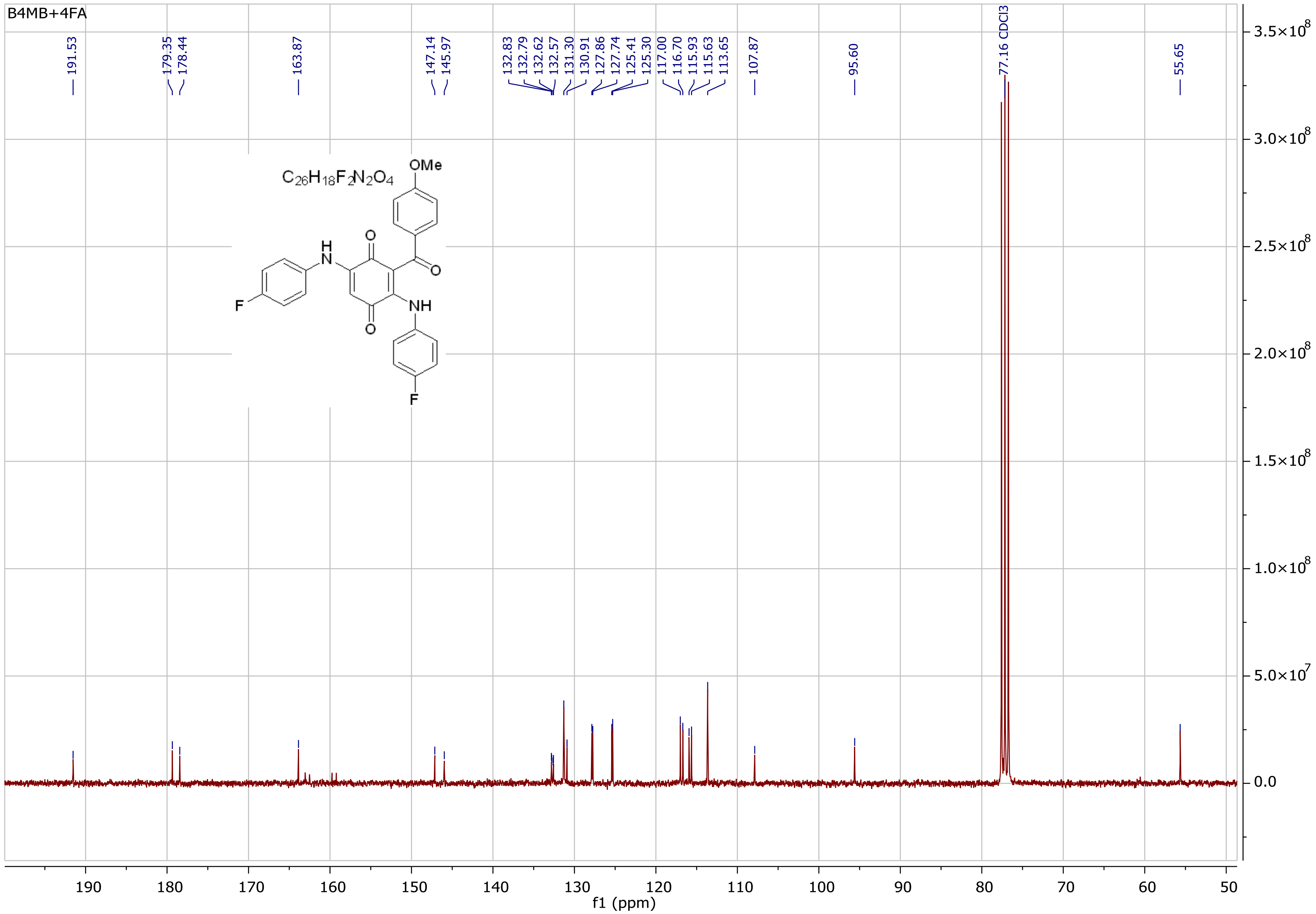

B4MB+4FA
191.53
179.35
178.44
163.87
147.14
145.97
132.83
132.79
132.62
132.57
131.30
130.91
127.86
127.74
125.41
125.30
117.00
116.70
115.93
115.63
113.65
107.87
95.60
77.16 CDCl3
55.65
C26H18F2N2O4
OMe
H
N
O
F
NH
f1 (ppm)

3-(Furan-2-carbonyl)-2,5-bis-[(4-fluorophenyl)amino]-1,4-benzoquinone **2p** **^{1}H NMR, ^{13}C NMR and HMBC**

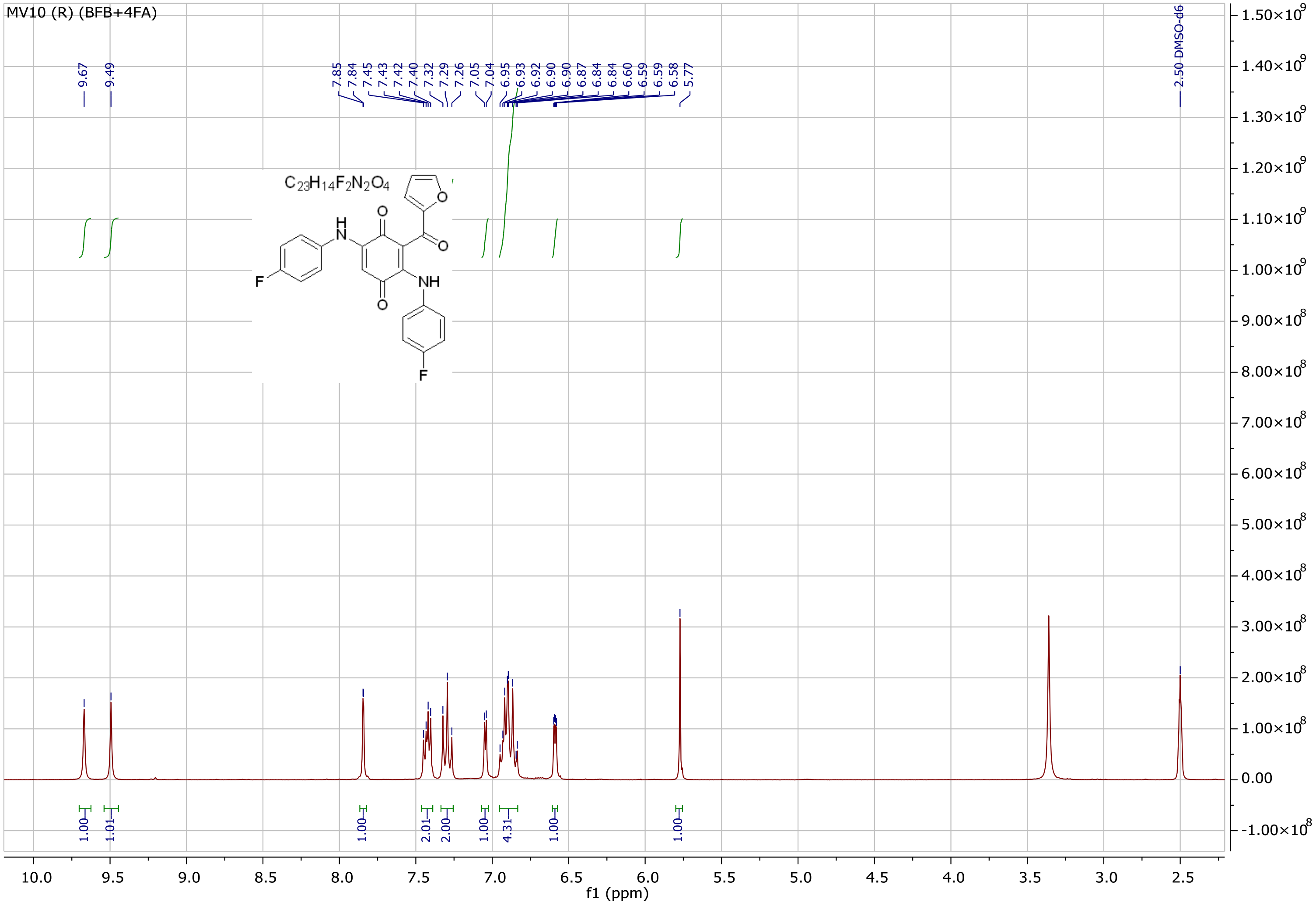

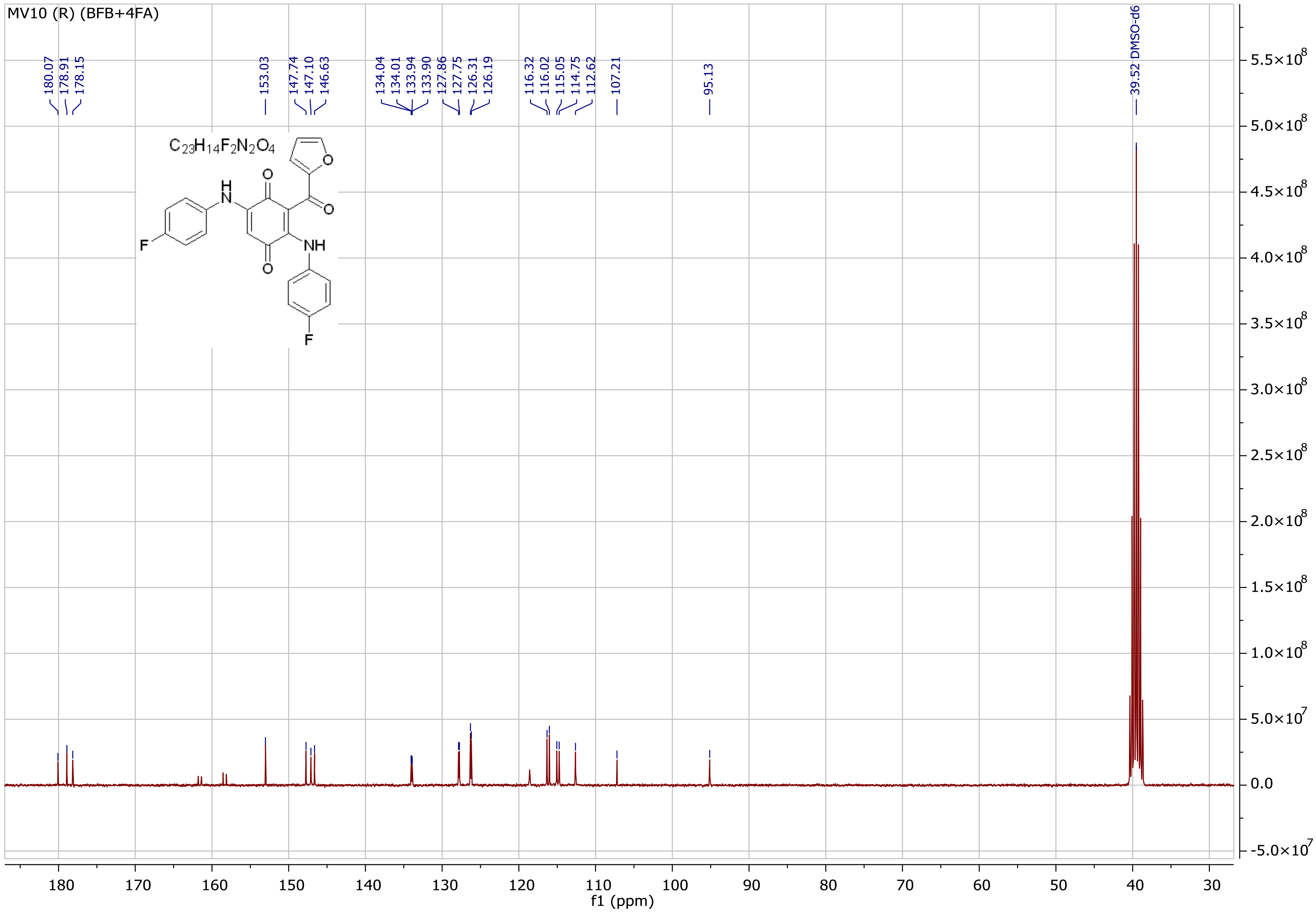
MV10 (R) (BFB+4FA)
180.07
178.91
178.15
153.03
147.74
147.10
146.63
134.04
134.01
133.94
133.90
127.86
127.75
126.31
126.19
116.32
116.02
115.05
114.75
112.62
107.21
95.13
39.52 DMSO-d6
C23H14F2N2O4
H
N
O
O
NH
F
F
O
O
f1 (ppm)
180 170 160 150 140 130 120 110 100 90 80 70 60 50 40 30
5.5×10^8
5.0×10^8
4.5×10^8
4.0×10^8
3.5×10^8
3.0×10^8
2.5×10^8
2.0×10^8
1.5×10^8
1.0×10^8
5.0×10^7
0.0
-5.0×10^7

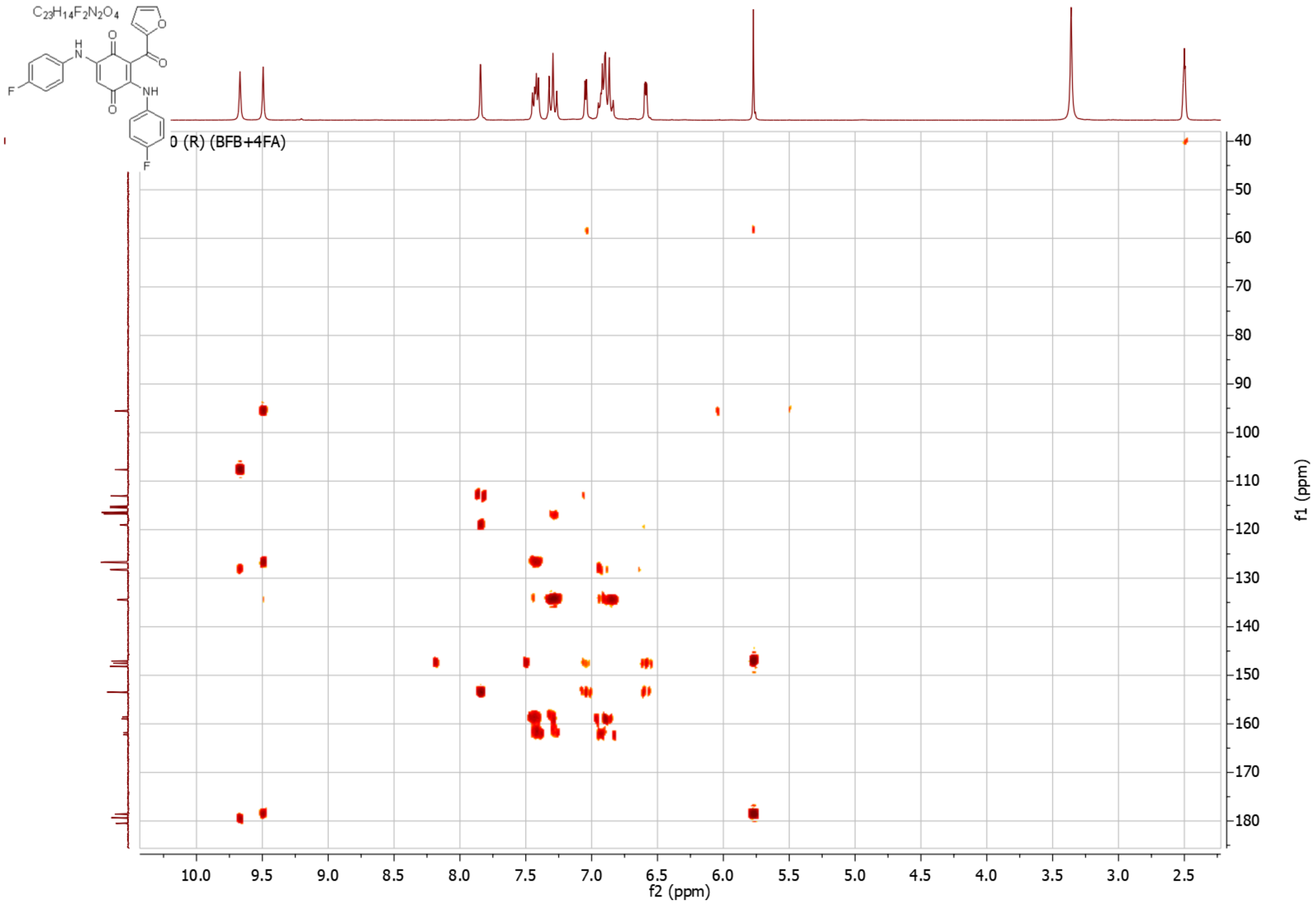
C23H14F2N2O4
0 (R) (BFB+4FA)
10.0 9.5 9.0 8.5 8.0 7.5 7.0 6.5 6.0 5.5 5.0 4.5 4.0 3.5 3.0 2.5
f2 (ppm)
40 50 60 70 80 90 100 110 120 130 140 150 160 170 180
f1 (ppm)
